# A Systematic Review of Systematic Reviews on the Epidemiology, Evaluation, and Treatment of Plantar Fasciitis

**DOI:** 10.3390/life11121287

**Published:** 2021-11-24

**Authors:** Hye Chang Rhim, Jangwon Kwon, Jewel Park, Joanne Borg-Stein, Adam S. Tenforde

**Affiliations:** 1 MetroWest Medical Center, Tufts University School of Medicine, Framingham, MA 01702, USA; hr233@cornell.edu; 2 Department of Physical Therapy, University of Delware, Newark, DE 19716, USA; jkwon@udel.edu; 3 Johns Hopkins Bloomberg School of Public Health, 615 N Wolfe St, Baltimore, MD 21205, USA; jpark299@jhu.edu; 4 Department of Physical Medicine and Rehabilitation, Harvard Medical School, Boston, MA 02115, USA; jborgstein@partners.org; 5 Spaulding Rehabilitation Hospital, Charlestown, MA 02129, USA

**Keywords:** plantar fasciitis, plantar fasciopathy, systematic review

## Abstract

The number of systematic review and meta-analyses on plantar fasciitis is expanding. The purpose of this review was to provide a comprehensive summary of reviews on the topic pertaining to plantar fasciitis, identify any conflicting and inconsistent results, and propose future research direction. A qualitative review of all systematic reviews and meta-analyses related to plantar fasciitis up to February 2021 was performed using PubMed, Embase, Web of Science, and the Cochrane Database. A total of 1052 articles were initially identified and 96 met the inclusion criteria. Included articles were summarized and divided into the following topics: epidemiology, diagnosis, and treatment. While the majority of reviews had high level of heterogeneity and included a small number of studies, there was general consensus on certain topics, such as BMI as a risk factor for plantar fasciitis and extracorporeal shockwave therapy as an effective mode of therapy. A qualitative summary of systematic reviews and meta-analyses published on plantar fasciitis provides a single source of updated information for clinicians. Evidence on topics such as the epidemiology, exercise therapy, or cost-effectiveness of treatment options for plantar fasciitis are lacking and warrant future research.

## 1. Introduction

Feet play an important role in posture and ambulation, and it has been reported that the prevalence of foot pathologies range between 61 and 79% and contribute to negative impact on quality of life [1]. Specifically, plantar fasciitis is a common musculoskeletal injury affecting individuals across ages and activity levels, and the condition is estimated to account for over 1 million physician visits annually in the United States [2,3,4,5]. Among athletes, the condition is especially prevalent in runners, affecting up to 17.4% of the running population [6]. Despite its name, plantar fasciitis is considered a degenerative pathology rather than a primary inflammatory condition [7]. As such, akin to how researchers and clinicians have adopted the use of “tendinopathy” in place of “tendinitis”, the terms “fasciosis” or “fasciopathy” are increasingly used in the literature to refer to the condition [8,9]. Further, other reports may discuss evaluation and treatment of common symptoms to the condition such as “plantar heel pain” [10,11]. The pathology is characterized by pain in the medial heel that is exacerbated by weight-bearing activity, as well as after periods of rest or non-weight bearing [12]. The injury is often chronic with typical symptoms lasting more than a year [13,14].

The importance in understanding this condition is reflected by a marked increase in published plantar fasciitis research. In a recent systematic review of trends in foot and ankle literature, plantar fasciitis was the second-most commonly published topic across five high-impact general medicine journals from 2000–2017 [15]. A PubMed search of “Plantar Fasciitis” returns over 1600 results in February 2021, with 636 items published in years 2015 to 2021. In particular, the number of systematic reviews and meta-analyses across topics within plantar fasciitis is ever-increasing; however, the results of these studies are conflicting and inconsistent which presents a challenge for physicians to interpret the clinical utility of these studies.

Systematic reviews of systematic reviews are being performed increasingly to summarize and evaluate current evidence within specific areas and can produce a broader overview of the current state of knowledge than more focused specific systematic reviews [16]. Therefore, the purpose of this qualitative review is to evaluate currently available evidence on plantar fasciitis through synthesizing and analyzing a large body of systematic reviews and meta-analyses published on the topic. The resulting summary aims to provide a detailed summary of evidence to clinicians that may assist in clinical decision making. Additionally, the review highlights gaps in knowledge of the topic of plantar fasciitis and guide future research.

## 2. Materials and Methods

### 2.1. Systematic Review Regisrtation

The protocol for this systematic review was registered at PROSPERO: CRD42021236673.

### 2.2. Search Strategy

PubMed, Embase, Web of Science, and the Cochrane Database of Systematic Reviews were searched for systematic reviews and meta-analyses published up to 7 February 2021. The search terms included plantar fasciitis, plantar heel pain, or plantar fasciopathy in combination with systematic review or meta-analysis. The detailed search strategy can be found in Supplementary Material Figure S1. The reference lists of the retrieved systematic reviews were also screened as a secondary search. Two authors (JK and JP) carried out the search independently to prevent any selection bias. After independently examining the titles and abstracts of the identified articles, these two authors compared their lists of included and excluded papers. Any disagreement during this process was resolved by consensus or discussion with the third author (HCR).

### 2.3. Inclusion Criteria

All systematic reviews or meta-analyses pertaining to the topic of plantar fasciitis (e.g., risk factors, diagnosis, or treatments) were eligible for inclusion. Reviews on plantar fasciopathy or fasciosis were included because these terms were used interchangeably with plantar fasciitis. Further, reviews on plantar heel pain were included since some authors did not make strict distinction between the terms plantar heel pain and plantar fasciitis. Systematic reviews or meta-analyses that investigated broader conditions such as foot and ankle or lower extremity were included as long as two or more studies on plantar fasciitis were analyzed or discussed as separate sections. If reviews were updated by the same authors or research group, only the most recent review was included.

### 2.4. Exclusion Criteria

Narrative reviews were excluded. The articles not published in English and abstracts were also excluded because it was difficult to assess the methodological quality of those systematic reviews.

### 2.5. Data Extraction

The Cochrane PICO (Patient, Population or Problem; Intervention; Comparison; and Outcome) components [17] were identified with consensus between the two authors (HCR and JK). The following data were also independently extracted by these two authors: (1) the list of first author and year of publication; (2) the type of plantar fasciitis topic; (3) the number and type of included studies in the systematic review; (4) the results of meta-analyses if present (4) summary findings of the systematic review; (5) limitations of the systematic review.

### 2.6. Methodological Quality Evaluation

The methodological quality of the included systematic reviews and meta-analyses was evaluated using the Assessing the Methodological Quality of Systematic Review 2 (AMSTAR 2) checklist [18]. Two authors (JK and JP) independently assessed the quality of included reviews using 16 items on this checklist. Unlike the original AMSTAR instrument, which was used to derive an overall score, the authors who revised AMSTAR and proposed AMSTAR2 recommended that users should define critical domains and rate overall confidence in the results of a systematic review based on the weakness of either critical or non-critical domains. The AMSTAR2 critical domains proposed by these original authors were adopted to evaluate the methodological quality of the included reviews. These critical domains included protocol registration, adequacy of the literature search, provision of a list of excluded studies and justification for exclusion, risk of bias assessment, appropriateness of meta-analysis, consideration of risk of bias for interpretation of the results, and assessment/impact of publication bias. If there was no or one non-critical weakness, the overall confidence in the results of the review was rated high; more than one non-critical weakness, moderate; one critical weakness with or without non-critical weakness, low; and more than one critical weakness without or without non-critical weakness, critically low. Any disagreement with the assessment was resolved by consensus or discussion with the third author (HCR).

## 3. Results

### 3.1. Eligible Studies

**Figure 1:**
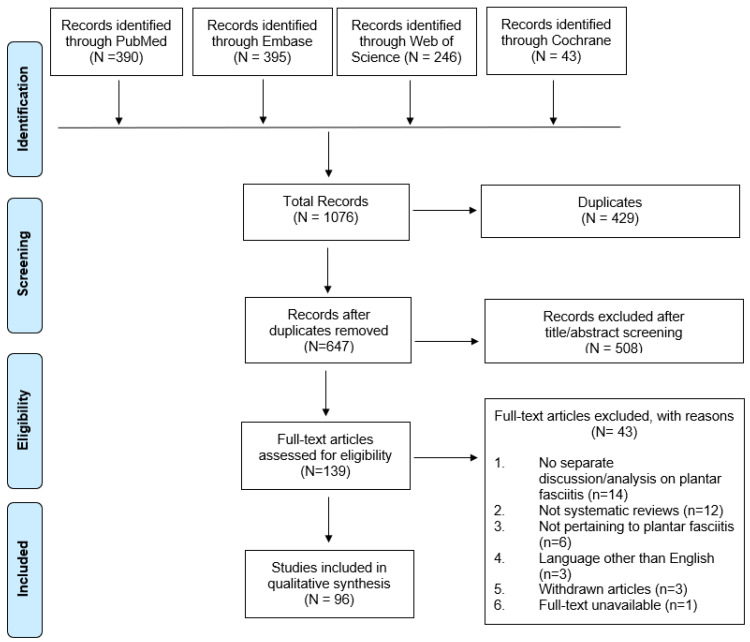
PRISMA (Preferred Reporting Items for Systematic reviews and Meta-Analyses) flowchart showing selection of articles pertaining to plantar fasciitis for qualitative analysis.

A total of 1052 articles were identified through the initial search. After removing 389 duplicates and screening 663 studies through the title and abstracts, 139 articles were selected for full-text review. 43 articles were excluded because (1) 14 reviews did not have separate discussion or analysis on plantar fasciitis; (2) 12 articles were not systematic reviews; (3) 6 reviews did not include any study related to plantar fasciitis; (4) 5 reviews included only one study related to plantar fasciitis; (5) 3 reviews were written in languages other than English; (6) 2 articles were withdrawn; and (7) 1 full-text article could not be retrieved. As a result, a total of 96 systematic reviews were included in this review. This search process is presented in the PRISMA flow chart (Figure 1).

### 3.2. Methodological Quality Evaluation

Using the AMSTAR2 criteria, among the 96 systematic reviews included in this review, the overall confidence in the results of 72 reviews (75%) was critically low, 17 reviews (17.7%) low, 3 reviews (3.1%) moderate, and 4 reviews (4.2%) high. Two critical domains that downgraded the overall confidence of the included reviews were failure to provide a list of excluded studies (82.3%) and lack of protocol registration (69.8%). The quality assessment of individual systematic review based on the AMSTAR2 criteria can be found in Appendix A Table A1.

### 3.3. Epidemiology

#### 3.3.1. Prevalence/Incidence

One review summarized the prevalence and incidence of plantar fasciitis [19]. Plantar fasciitis is most common between 40 and 60 years of age and contributes to 15% of foot injuries in general population without gender difference. The conditions may affect both athletic and non-athletic populations, but the incidence is higher among runners.

#### 3.3.2. Risk Factors

- Body mass index (BMI): The association between BMI and plantar fasciitis has been examined in multiple reviews. Butterworth et al. examined the association between body mass index (BMI) and musculoskeletal foot disorders and included 12 studies (nine matched case-control and three cross-sectional) on plantar fasciitis [20]. The authors found that while BMI was not associated with plantar fasciitis in the athletic population, there was evidence to support such an association in the non-athletic population [20]. Likewise, Franceschi et al. reported a strong association between increased body weight and plantar fasciitis based on four observational studies [21]. These results have been supported by other reviews that specifically investigated risk factors of plantar fasciitis, especially in non-athletic population [22,23,24].
- Weightbearing activities: Waclawski et al. specifically focused on adult workers and explored the association between weight-bearing activities (such as walking or standing) and plantar fasciitis [25]. The authors identified four studies and found that there was low-quality evidence to support an association between weight-bearing tasks and plantar fasciitis.
- Muscle function and size: Osborne et al. investigated muscle function and muscle size differences between those with and without plantar fasciitis [26]. Seven studies were included in this review, and the strength of muscle groups including hallux plantar flexion, lesser toe plantar flexion, ankle dorsiflexion, ankle inversion, and ankle eversion were lower in patients with plantar fasciitis. Foot muscle volume was also smaller in people with plantar fasciitis. However, there was no significant difference in calf muscle endurance between people with and without plantar fasciitis. The authors cautioned that these results are from studies with GRADE ratings suggesting strength of evidence rated as very low, and they concluded the role of muscle strength warrants further investigation.
- Kinematics: Mousavi et al. focused on distance runners with aims to identify kinematic risk factors for lower limb tendinopathy [27]. The authors included two studies that compared kinematic data of runners suffering from plantar fasciitis with healthy runners and found no significant difference between the two groups. However, another review concluded that decreased ankle dorsiflexion and decreased first metatarsophalangeal joint extension were weakly associated with plantar fasciitis [22]. The most recent review by Hamstra-Wright et al. investigated 16 studies focusing on physically active individuals and identified that increased plantarflexion range of motion was a risk factor [24]. Other biomechanical aspects such as ground reaction forces did not have sufficient studies to draw conclusions.
- Others: A review published in 2006 included 16 articles and found that the presence of a calcaneal spur were consistently associated with plantar fasciitis, and weaker associations for increased age, and prolonged standing [22]. These findings were echoed by van Leeuwen et al. who identified 51 articles, evaluated a total of 104 variables, and conducted a meta-analysis for 12 variables [23]. These authors found that patients with plantar fasciitis were more likely to have increased plantar fascia thickness, hypoechogenicity, and subcalcaneal spurs primarily identified on ultrasound and X-ray.

### 3.4. Diagnosis

Five reviews investigated imaging tools of diagnosing plantar fasciitis [28,29,30,31]. McMillan et al. identified 23 studies examining all diagnostic imaging features associated with plantar fasciitis [28]. Among different imaging modalities such as ultrasonography, MRI, and plain radiography (i.e., X-ray), ultrasonography was most widely, and primary diagnostic criteria from imaging was evaluating plantar fascia thickness. Meta-analysis showed that patients with plantar fasciitis had 2.16 mm thicker plantar fascia than controls and tended to have absolute plantar fascia thickness values exceeding 4.0 mm. Two studies published more recently focused on evaluating the effectiveness of using ultrasound imaging and confirmed that ultrasound could be an accurate and reliable imaging tool for diagnosing plantar fascia, improving accuracy in the delivery of interventions, and monitoring improvement after interventions [29,30]. Fusini et al. investigated clinical applications of real-time sonoelastography in tendon disorders and included 6 studies on plantar fasciitis [31]. The authors found that patients with symptomatic plantar fasciitis had measures suggesting “softer” plantar fascia, and sonoelastography could detect positive findings for plantar fasciitis in symptomatic patients with normal ultrasound findings.

McMillan et al. investigated the association between subcalcaneal spurs and plantar fasciitis [28]. Meta-analysis of seven studies demonstrated that patients with plantar fasciitis were 8 times more likely to demonstrate radiographic evidence of subcalcaneal spurs than controls. This association became stronger (odds ratio = 16.11) after excluding non-blinded studies. The authors suggested a causal relationship could not be established and urged that further research would be necessary to better understand the role of calcaneal spur and the development of plantar fasciitis.

Petragila et al. evaluated diagnostic strategies for athlete and non-athletes separately, and identified one of 9 included studies made a distinction between athletes and non-athletes [32]. The authors suggested that imaging studies could be useful when symptoms are equivocal or to differentiate other possible causes of heel pain but formal finding specific to athlete populations were poorly characterized.

### 3.5. Treatments

#### 3.5.1. Corticosteroid

Corticosteroid has been used for the treatment of plantar fasciitis as it can reduce inflammation, fibroblast proliferation and ground substance proteins, which have been thought to play roles in pathogenesis of plantar fasciitis [11].

There were four reviews identified that evaluated corticosteroid injection for treating plantar fasciitis [11,33,34,35]. The earliest review including four randomized controlled trials (RCTs) concluded that compared to placebo, corticosteroid injection was more effective in relieving pain measured by visual analogue scale (VAS) at one month but not over longer intervals [33]. Chen et al. included nine RCTs and found that corticosteroid injection provided better pain relief at 1–1.5 month and 2–3 months compared to other interventions [34]. These pooled results compared corticosteroid injection with non-invasive treatments that included shockwave, physical therapy, insole, and NSAIDs. Therefore, significant heterogeneity in the comparison group requires a cautious interpretation. Subgroup analysis revealed that corticosteroid injection was more effective in pain reduction than physical therapy at 1–1.5 month. However, no significant difference was found between corticosteroid injection and other non-invasive treatments. David et al. conducted a more comprehensive review including 36 RCTs and 3 quasi-RCTs [11]. The authors found low quality evidence for corticosteroid reducing pain for up to one month compared to placebo or no treatment without sustained benefits longer term. For other comparisons, evidence was imprecise and at high risk of bias. Among 21 trials that reported adverse events, two ruptures of plantar fascia, three injection site infections, and 27 minor adverse events such as post-injection pain were reported. The most recent review included 47 RCTs that compared corticosteroid injection to any comparator [35]. In the short term (zero to six weeks), corticosteroid injection was more effective than autologous blood injection and foot orthoses (low quality evidence) while in the long term (13 to 52 weeks), PRP injection and dry needling was more effective in terms of reducing pain (very-low quality evidence). Also, placebo injection had similar efficacy to corticosteroid injection in pain reduction in the short and medium terms (moderate quality evidence). For functional improvement, corticosteroid injection was more beneficial than physical therapy in the short term (low quality evidence).

Li et al. compared ultrasound- versus palpation-guided corticosteroid injections [36]. The review included five RCTs and found that ultrasound-guided injection provided better outcomes in terms of VAS scores, tenderness threshold, response rate, plantar fascia thickness, and hypoechogenicity. David et al. analyzed the same five studies and reported a similar finding for pain reduction but concluded that the evidence was very low quality because all five RCTs were at high risk of bias [11].

#### 3.5.2. Platelet-Rich Plasma (PRP)

PRP or other autologous blood products are harvested from a patient’s own peripheral blood and contain storage pools of growth factors such as platelet-derived growth factor, transforming growth factor, vascular endothelial growth factor. The rationale behind using such platelet-rich preparations is the idea that the additional platelets may increase the growth factors at the injury site and enhance the healing process [37].

Eight reviews examined the efficacy of PRP over control or in comparison with other therapies for plantar fasciitis [37,38,39,40,41,42,43,44]. Four studies performed qualitative review without meta-analysis [37,39,43,44]. While Vannini et al. found that the evidence was inconclusive [37], three reviews concluded that PRP showed promising results without major complications [39,43,44]. Notably Vannini et al. included two studies, and level of evidence was determined as low for three reviews. Ling et al. conducted a meta-analysis of ten RCTs comparing PRP with other treatments [40]. Of these, nine used steroid injection as control regimen, and the remaining one used whole blood. One study concluded PRP showed a significant decrease in VAS scores and two studies demonstrated improvement in AOFAS score at 12 months, but all studies showed no improvements over earlier intervals [40]. Yu et al. evaluated 13 RCTs and conducted separate meta-analyses for PRP versus corticosteroid and PRP versus placebo [41]. While the authors concluded that PRP is not superior to corticosteroid, there was some evidence that PRP is better than placebo.

#### 3.5.3. PRP vs. Corticosteroid

There were ten reviews comparing the efficacy of PRP (or autologous whole blood) versus corticosteroids. The oldest review published in 2016 by Tsikopoulos et al. included only 3 RCTs and reported that corticosteroid was slightly more effective than autologous whole blood in the short term (2–6 weeks), but not in the medium term (24–26 weeks) [45]. Singh et al. who performed meta-analysis of ten prospective studies concluded that PRP injections were associated with improved VAS scores and AOFAS scores at 3 months but not at 6 months [46]. Yang et al. analyzed nine RCTs and found that PRP resulted in more pain reduction than corticosteroid at 6 months, but not at 1 month and 3 months, and there was no difference in AOFAS scores between two treatments [47]. Chen et al. included 12 RCTs and 4 quasi-experimental studies and concluded that corticosteroid was more effective in reducing pain at 1.5 and 3 months than whole blood while PRP showed more benefit than corticosteroid at 6 months [48]. Similar to Yang et al., Chen et al. also found no difference in AOFAS scores between two treatments at any time point.

The remaining reviews, all published in 2020, used different inclusion criteria and search periods which resulted in conflicting results [49,50,51,52,53,54]. Of these, four reviews combined RCTs with non-RCTs [49,50,51,52]. Huang et al. included 10 studies, but in their analysis, they ended up pooling only eight RCTs and concluded that PRP provided long-term (≥6 months) improvement in AOFAS scores over corticosteroids, but no difference in pain VAS scores were observed between PRP and corticosteroids [49]. Mohammed et al. included 10 prospective studies and found that PRP showed greater VAS score reduction compared to corticosteroid at 3 and 6 months [50]. Alkhatib et al. included 4 prospective cohort studies and 11 RCTs and concluded that PRP was associated with higher AOFAS score at 6 months and lower VAS scores at 6 and 12 months [52]. Hohmann et al. also analyzed 15 level 1 and 2 studies, and while 13 studies overlapped with those included in the review by Alkhatib et al., two were unique to Hohmann’s study. Hohmann et al. found that PRP showed improvements in VAS scores at 3, 6, and 12 months and AOFAS scores at 6 and 12 months [51]. The remaining two reviews restricted inclusion criteria to only analyzing RCTs, but differed in defining PRP and autologous blood products [53,54]. Tseng et al. combined PRP with autologous whole blood, Hurley et al. included only PRP [53,54]. The results were also different, as Hurley et al. found that PRP offered benefits in terms of VAS score reduction at 1–1.5, 3, 6, and 12 months and AOFAS score improvement at 6 and 12 months over corticosteroid; [53] however, Tseng et al. found no difference between autologous blood-derived products (e.g., PRP and whole blood) and corticosteroid as measured by VAS or AOFAS scores at all time points [54]. It is important to note that even though those authors who limited their analysis to PRP, heterogeneity in PRP such as different concentrations of platelets, leukocytes, growth factors as well as variation in centrifugation speed and time were seen across the studies they included [46,47,49,50,51,52,53].

#### 3.5.4. Extracorporeal Shockwave Therapy (ESWT)

Shockwaves are pressure pulses of microsecond duration and have peak pressures of 35–120 MPa. There are two types of ESWT used in clinical practice, focused EWST and radial ESWT. Focused EWST can concentrate shockwaves at the target site and penetrate deeper than radial ESWT. The proposed mechanisms for the effect of ESWT include hyperstimulation analgesia and stimulation of neovascularization and collagen synthesis in degenerative tissues [55,56].

Two reviews investigated the effect of shockwave therapy in soft tissue [55] or musculoskeletal [57] conditions and discussed plantar fasciitis separately in their reviews. Speed conducted a systematic review including 12 RCTs without meta-analysis and concluded that high-dosed focused ESWT and radial shockwave have benefits in pain reduction at 12 weeks [55]. Al-Abbad et al. performed a meta-analysis of seven studies and showed an overall reduction in plantar fascia thickness after ESWT at 4 weeks, 6 weeks, 3 months, and 6 months, and a greater reduction was found when radiologic guidance was used [57].

Ogden et al. published a systematic review in 2002 including 20 studies and suggested that ESWT could be considered prior to surgical intervention and that it is preferrable to corticosteroid injection which has a potential risk of subsequent plantar fascia rupture and lacks long-term safety data regarding multiple injections and possible systemic effects [58]. Thomson et al. conducted a meta-analysis of 6 RCTs in 2005 and reported that ESWT have benefits in morning pain reduction over placebo at 12 weeks, but this effect was not observed when the authors limited to studies rated as high-quality RCTs in the analysis [59]. In more recent three overlapping meta-analyses of RCTs [56,60,61], ESWT showed greater reduction in VAS scores and success rate of improving heel pain by 60% over placebo when taking first steps and during daily activities. The most recent review by Sun et al. included 13 level 1 or level 2 studies that compared ESWT with other therapies and found greater success rate, higher Roles and Maudsley scores, greater reduction in VAS scores, decreased return to work time, and less complications in those treated with ESWT compared to other interventions. Notably the alternative treatments in the review included placebo, ultrasound therapy, and even endoscopic plantar fasciotomy, thus amounting to significant heterogeneity [62].

Five reviews assessed the efficacy of different energy levels used in either focused or radial ESWT for treating plantar fasciitis [63,64,65,66,67]. Li et al. limited their analysis to 5 RCTs examining high-energy ESWT (energy flux density > 0.2 mJ/mm^2^) and found that among those suffering from plantar fasciitis over 6 months with failed conservative managements, ESWT provided higher clinical success than placebo [63]. Yin et al. divided ESWT into low and high levels using an energy flux density cutoff of 0.2 mJ/mm^2^ and found that low intensity ESWT had higher success rates and greater functional improvement as measured by Roles and Maudsley score. It is important to note, however, that there were only two studies that explored high-intensity ESWT [64]. Dizon et al. categorized ESWT into three groups (low, energy flux density < 0.1 mJ/mm^2^; medium, 0.1–0.2 mJ/mm^2^; high, >0.2 mJ/mm^2^) included 11 high-quality RCTs. They found that ESWT was in overall effective in reducing VAS scores and improving Roles and Maudsley scores at 12 weeks. However, there were only 2 or 3 studies in each energy level group. Therefore, the authors’ claim that moderate- or high-intensity ESWT were effective in reducing pain should be interpreted with caution [65]. Chang et al. divided focused ESWT into three subgroups, low intensity (energy flux density ≤ 0.08 mJ/mm^2^), medium intensity (energy flux density 0.08–0.28 mJ/mm^2^), and high intensity (energy flux density ≥ 0.28 mJ/mm^2^) while treating radial ESWT as a separate group. The results of pairwise meta-analysis showed that medium and high-intensity focused ESWT yielded higher success rates and pain reduction than the placebo. The network meta-analysis demonstrated the opposite result with the pairwise meta-analysis, reporting radial ESWT to be the most optimal therapy. The authors noted that due to the limited number of included studies and heterogeneity across the studies led to inconsistent results [66]. The most recent review by Wang et al. included more studies in meta-analysis than the other four reviews, separated articles into three groups as reported by Dizon et al., and further divided the studies according to follow-up durations. While high-intensity and low-intensity ESWT showed some benefit, the authors suggested that medium-energy ESWT provided greater success rates and pain reduction that focused ESWT may be more effective than radial ESWT [67].

Two reviews compared ESWT with corticosteroid injections [68,69]. Xiong et al. included 6 RCTs in their systematic review and concluded that ESWT may offer more pain reduction at 3 months based on two studies [68]. Li et al. conducted a meta-analysis of nine RCTs including six studies that were analyzed by Xiong et al. Li et al. divided ESWT into high and low intensity using 0.2 mJ/mm^2^ as the cutoff for energy flux density and found that high intensity ESWT was more effective than corticosteroid injection while corticosteroid injection was more effective than low intensity ESWT in terms of pain relief and success rate within 3 months [69].

Another study by Li et al. compared ESWT with ultrasound therapy. The authors conducted a meta-analysis with 5 RCTs and concluded that ESWT showed greater improvement in VAS than ultrasound therapy, but there was no significant difference in the AOFAS score, foot function index, and plantar fasciitis pain and disability scale [70].

Roerdink et al. specifically examined complications of ESWT in plantar fasciitis. The authors included 39 studies and concluded that while there may be common side effects such as pain during treatment and transient erythema, complications during the first year of follow-up are highly unlikely and concluded that both low- and high-dose ESWT are safe for treating plantar fasciitis [71].

#### 3.5.5. Mechanical Treatments

Mechanical treatments are commonly used in various clinical settings in the treatment of plantar fasciitis. For purposes of this review, we define this as interventions including insoles, taping, various orthoses, night splints, and specialized shoes. They are often utilized by patients due to accessibility, ease of use, and low risk of adverse events.

- Insoles: Six reviews investigated the effects of insoles in the treatment of plantar fasciitis with mixed findings. Lee et al. performed a meta-analysis of 6 RCTs and cohort studies evaluating the effect of insole interventions in patients with a clinical diagnosis of plantar fasciitis. The authors found significant improvements in both pain and function in patients using insole orthotic devices in short (less than 6 weeks), medium (6–12 weeks), and long terms (more than 12 weeks) [72]. Two systematic analyses similarly reported favorable findings for insole use [73,74]. In contrast, four studies reported limited or no benefits from insoles compared to other interventions [75,76,77,78]. Hawke et al. conducted a meta-analysis of 5 RCTs and found significant improvements in function, but not pain, for custom versus sham orthotics after 12 weeks. They also concluded that custom orthotics were less effective in improving pain and function than treatments consisting of manipulation, mobilizations, and stretching at 2 weeks but there were no differences after 2 weeks between the groups [75]. Whittaker et al. conducted a similar systematic review with meta-analyses of 19 RCTs and found significant differences in pain relief when comparing sham versus true orthotics; conversely, potential benefits were observed in sham condition in the medium term (6–12 weeks), with differences not meeting minimally clinically significant values when back-transformed to the pain subscale of the Foot Health Status Questionnaire [76]. Rasenburg et al. reported similar findings and concluded that insoles were not superior for improving pain, function, or self-reported recovery compared to other conservative interventions across 20 RCTs [77]. Healy et al. also suggested that the lack of high-quality studies precluded any conclusions about orthotic interventions’ effectiveness and cost-effectiveness in treating plantar fasciitis [78].
- Four studies specifically investigated the effects of custom versus prefabricated insole orthotics [73,75,76,77]. Three of four found minor or no improvements in pain and function for prefabricated versus custom orthoses, custom versus sham orthoses, and prefabricated versus sham orthoses. Three studies also examined the effects of orthotics versus night splints [75,76,77]. All three reported no differences in pain reduction, although Hawke et al. found a significant improvement with combined use versus individual use of either device.
- Taping: Two systematic reviews explored the use of taping as an intervention for patients with plantar fasciitis [79,80]. Podolsky et al. reported Low Dye taping was the most commonly used technique across eight different studies followed by calcaneal taping. While taping may be a beneficial technique in improving pain in the short term (less than 1 week), authors could not draw conclusions for longer term outcomes as the longest follow-up was only one week among the included studies [79]. Van de Water et al. reported similar findings, with limited evidence favoring Low Dye and calcaneal taping versus sham interventions for improving pain, but not function, after 1 week [80]. The authors also noted that a combined intervention of taping and stretching may be superior to stretching alone. Both studies could not perform quantitative synthesis due to the heterogeneity of methods in their included studies.
- Mixed treatment: Schuitema et al. conducted a systematic review of 43 studies to investigate the effects of various commonly used mechanical interventions in the treatment of plantar fasciitis, including foot orthotics, taping, ankle foot orthoses, night splints, and specialized shoes [74]. The authors suggested that mechanical treatments overall may be beneficial in symptom reduction. However, the authors added that methodological limitations of included studies prevented them from making any comparisons between them regarding relative effectiveness and frequent use of non-mechanical co-interventions also confounds interpretation of role of each intervention.

#### 3.5.6. Needling Therapies

Needling therapies involve the insertion of fine needles into patients’ skin or other tissues by skilled practitioners. Acupuncture refers to a family of therapies based largely on Eastern medicine principles. Dry needling involves the insertion of needles into myofascial trigger points.

- Acupuncture: Two systematic reviews investigated the use of acupuncture in the treatment of plantar fasciitis [81,82]. Both concluded that acupuncture therapies were associated with symptom reduction over outcome measures obtained 1–6 months although each cited significant heterogeneity of methods amongst their included studies as a major limitation.
- Dry needling: Two studies investigated the use of dry needling in the treatment of plantar fasciitis [83,84]. Cotchett et al. conducted a systematic review of three quasi-experimental studies and found limited evidence favoring the use of dry needling and poor quality of included studies [83]. He et al. conducted a meta-analysis of 7 RCTs published seven years after Cotchett et al., and compared trigger point dry needling or acupuncture to other interventions [84]. The authors concluded that myofascial trigger point needling was associated with significantly greater reductions in pain in at 1, 6, and 12 months. However, they reported substantial heterogeneity of methods, poor quality, and small sample sizes inherent in their included studies.

#### 3.5.7. Low-Level Laser Therapy (LLLT)

LLLT is a form of photobiomodulation therapy that involves the use of wavelengths ranging from 620 nm^10^ to the infrared (820–904 nm) over the surface of patient’s skin. The proposed mechanisms include anti-inflammatory effect as well as stimulation of cell proliferation, microcirculation, vascular neoformation, and collagen production [85].

Two studies investigated the use ofLLLT in the treatment of plantar fasciitis [85,86]. Dos Santos et al. conducted a systematic review of sevenRCTs, of which four also included meta-analyses. The authors found that LLLT significantly improved pain and function and decreased plantar fascia thickness compared to other therapies, such as exercise. However, they reported a high degree of heterogeneity in the methodology and results of their included studies, as well as large variations in the treatment parameters of LLLT. Wang et al. performed a meta-analysis of six RCTs investigating the effectiveness of LLLT in reducing pain and reported favorable findings for the use of LLLT at study end point (3 months).

#### 3.5.8. Exercise Therapy

- Strengthening exercise: Only one study examined the effects of strengthening interventions on plantar fasciitis [87]. Huffer et al., who conducted a systematic review of seven studies, classified strengthening interventions into three distinct categories—minimalist running shoe intrinsic foot muscle (IFM) strengthening, IFM foot exercises, and plantar aponeurosis loading. While the authors found that these minimalist running shoes and toe flexion against resistance may improve intrinsic foot musculature in asymptomatic populations, high-load plantar fascia resistance training has not been shown to change plantar fascia thickness. Therefore, reviews determined no definitive conclusions regarding the benefits of strengthening interventions in patients with plantar fasciitis could be made.
- Stretching: Three studies investigated the effects of stretching in the treatment of plantar fasciitis [88,89,90]. Sweeting et al. conducted a systematic review of six studies comparing stretching with other conservative treatments or without intervention/natural history [88]. Patients who received stretching interventions seemed to improve over time in both pain and function, especially in the first two weeks to four months. However, the reviewers concluded that there were no significant differences between stretching and other interventions. They also noted that their study was limited by the large degree of heterogeneity in techniques, dosages, and comparison groups between individual studies, which made it difficult to comment on the relative effectiveness of different stretching regimens. Woitzik et al. conducted a systematic review of six RCTs investigating the effects of exercise on clinical outcomes in various soft tissue injuries of the lower extremities [90]. Of these, two studies that focused on plantar heel pain provided preliminary evidence supporting the use of static plantar fascia stretching in improving pain and function, but no support for static calf stretching. These findings were largely consistent with report by Siriphorn et al. who conducted a systematic review with meta-analysis of eight RCTs and found that there was moderate quality evidence in favor of plantar fascia-specific stretching (PFSS) over the Achilles tendon or calf stretching (CS) reducing pain in the short term (less than 3 months [89].

#### 3.5.9. Manual Therapy

Manual therapies are interventions performed by clinicians directly on patients using their hands or other parts of their body, tools, and/or other modalities to apply force to tissue and joints. They include, but are not limited to, treatments such as soft tissue massage, joint mobilizations, manipulations, myofascial trigger point releases, and contract-relax stretching. This form of treatment is most commonly performed in physical therapy or chiropractic settings.

Five systematic reviews investigated the use and effectiveness of manual therapies on patients with plantar fasciitis [91,92,93,94,95]. Of these, three studies looked into the use of manual therapy in the treatment of various conditions that included plantar fasciitis. Brantingham et al. defined manipulative therapy as “all types, methods, modes, and techniques of mobilization and manipulation grades I–V” and included two studies that pertained to plantar fasciitis [91]. The authors concluded that there was moderate level of evidence supporting the use of manipulative therapies of the ankle and foot on patients with plantar fasciitis in the short term (1–5 weeks) when combined with multimodal or exercise therapies. Clar et al. evaluated 3 studies on plantar fasciitis and reported that there was inconclusive evidence favoring the use of trigger point therapy in the treatment of plantar fasciitis [92]. Piper et al. examined the effects of soft-tissue therapy in the treatment of musculoskeletal disorders and injuries of the upper and lower extremities. Based on two studies pertaining to plantar fasciitis, the authors concluded that myofascial release was effective in the management of plantar heel pain [93].

Two systematic reviews investigated the effectiveness of manual therapies specifically in the treatment of plantar fasciitis [94,95]. Fraser et al. performed a systematic review of seven RCTs comparing manual therapies such as including soft tissue mobilizations and joint mobilizations/manipulations compared with other intervention on outcomes including patient-reported pain, function, and pressure-pain thresholds. The authors found that manual therapy combined with stretching or strengthening led to greater improvements in function and pain pressure thresholds. They reported that patients’ pain seemed to improve over time across interventions. However, authors concluded that the effectiveness of one manual technique versus another could not be determined due to the heterogeneity of study designs and variety of manual techniques. Mischke et al. conducted a systematic review of eight RCTs and concluded that manual therapy—which they defined as an intervention where a therapist used their hands to perform treatment —may be effective in improving pain and function both in short term (<4 weeks) and over greater follow-up time (>4 weeks). Similar to Fraser et al., the authors of this study reported that their results should be interpreted with caution, due to the poor methodological quality heterogeneity in dosing, techniques, and outcome measures, and the frequent use of co-interventions secondary to manual therapy within included studies.

#### 3.5.10. Prolotherapy

Prolotherapy uses the injection of dextrose which is thought to induce local cell necrosis at the injection site which in turn initiates the body’s healing process with inflammation, granulation tissue formation, matrix formation and remodeling [96].

Sanderson et al. investigated effectiveness and safety of prolotherapy injections for managing lower limb tendinopathy and fasciopathy [96]. In their review, they included two studies on plantar fasciitis and found that prolotherapy may offer benefits in pain reduction; notably the included studies lacked a true control group because one study had case-series design and the other study compared prolotherapy with PRP.

#### 3.5.11. Iontophoresis

Iontophoresis refers to a iontophoretic transdermal controlled drug delivery system which delivers diverse compounds such as corticosteroids and NSAIDs in a controlled manner [97].

Clijsen et al. evaluated the effects of iontophoresis in the treatment of musculoskeletal disorders and included three studies on plantar fasciitis that measured outcomes at 1 month and 3 months [97]. However, the drugs used in the iontophoresis differed in these studies and findings across studies were conflicting. Therefore, the efficacy of iontophoresis for the treatment of plantar fasciitis remains unclear.

#### 3.5.12. Endoscopic Plantar Fasciotomy

Surgical management can be considered when symptoms persist despite efforts of non-operative management. Endoscopic plantar fasciotomy involves performing a release in the plantar fascia with or without spur removal, nerve decompression, or calcaneal drilling [98].

Two studies have examined the effectiveness of endoscopic plantar fasciotomy for plantar fasciitis [98,99]. Malahias et al. performed a systematic review of 15 studies and found that endoscopic plantar fascia release improved post-operative clinical outcomes with overall complication rate of 11.0% [98]. However, the few comparative studies with comparing endoscopic plantar fascia release to extracorporeal shockwave therapy (ESWT) or platelet-rich plasma (PRP) did not show superior outcomes [98]. The quality of studies included in the review for endoscopic surgery was low based on the GRADE guidelines. Indeed, 10 of the included studies were case series while only 2 studies were RCTs. Therefore, the authors concluded that there was weak evidence to recommend the use of endoscopic fascia release for the treatment of chronic plantar fasciitis.

Mao et al. performed a meta-analysis to quantify the effect of endoscopic plantar fasciotomy that included studies with minimum follow-up of two years [99]. While endoscopic plantar fascia release was associated with an increase of 34.2 points in the American Orthopaedic Foot and Ankle Society (AOFAS) Ankle-Hindfoot score postoperatively, this study also suffered from the low quality of the included studies. Thus, the authors concluded that the grade of recommendation was poor (grade C) based on the classification of Wright [99].

### 3.6. Comparative Evaluation of Multiple Treatments

Two reviews qualitatively analyzed multiple treatment options in the lower extremities. Sutton et al. included only studies that used multimodal care, defined as a treatment approach with at least two distinct therapeutic modalities. The authors identified two studies with low risk of bias for plantar fasciitis and suggested that a multimodal care including mobilization and stretching exercise may be helpful [100]. Yu et al. included 13 studies with low risk of bias and concluded that ultrasound or foot orthoses may be beneficial for patients with persistent plantar fasciitis while Low Dye taping may be not. Further, authors suggested that ESWT may not be helpful for recent (3 months or less) plantar fasciitis [101].

Five reviews focused on plantar fasciitis and qualitatively evaluated available treatment options [102,103,104,105,106]. In 1999, Atkins et al. concluded that there had been limited RCTs, and thus, robust scientific evidence for treatment efficacy was lacking [102]. Uden et al. analyzed 6 RCTs in 2011 and suggested that customized foot orthoses and corticosteroid injections may reduce pain associated with plantar fasciitis [103]. Assad et al. assessed 8 RCTs in 2016 and found that among various treatment options, autologous blood injection or PRP may be preferred for long term management [104]. Petragila et al. included eight studies and concluded that due to the heterogeneity among the studies, practice guidelines or best treatment algorithm could not be suggested [32]. Al-Boloushi et al. in 2019 also concluded that there was no definitive treatment guideline for plantar fasciitis but suggested that ESWT, botulinum toxin type-A injections, PRP, dry needling, and corticosteroid could be available non-surgical treatment options based on the 29 articles reviewed [105].

Six reviews included meta-analyses to quantitatively assess and compare treatment modalities for plantar fasciitis [10,106,107,108,109,110]. Salvioli et al. used a traditional pairwise meta-analysis to compare ESWT, laser therapy, orthoses, and stretching versus placebo. Except for stretching, the meta-analyses showed significant improvements in favor of intervention, but the quality of evidence was low due to moderate risk of bias of the included studies [106]. The other five reviews incorporated a network meta-analysis (NMA) which allows for comparisons of multiple treatment options and helps determine relative efficacy. A NMA published in 2015 including 7 RCTs and 3 quasi-experimental studies demonstrated that autologous blood products could be a preferred option at 3 months while ESWT was superior to autologous blood products at 6 months [107]. Tsikopoulos et al. conducted a NMA of 22 RCTs that investigated injection therapies for plantar fasciitis and found that in terms of pain relief, dehydrated amniotic membrane intervention was beneficial at 0–2 months while botulinum toxin-A was effective at 0–6 months. However, the result of dehydrated amniotic membrane intervention was based on one study, and therefore, should be interpreted with caution [108]. Li et al. included 19 RCTs investigating five different therapies, and their NMA showed that radial ESWT was the only intervention to result in pain reduction compared with placebo at 0 to 6 weeks [109]. Another group of researchers included 41 trials evaluating 8 different therapies in their NMA and found that ESWT consistently provided pain relief at 1 month, 3 month, and 6 months and ranked first of being the optimal regimen according to the surface under cumulative ranking curve [110]. The most recent NMA by Babatunde et al. concluded that while corticosteroid injections and ESWT were ranked most likely to be beneficial in terms of pain or function improvement in short, medium and long terms, current evidence is still ambiguous regarding which therapy is most effective for the management of plantar fasciitis [10].

### 3.7. Psychosocial Variables

Drake et al. conducted a systematic review of 5 studies to investigate the effects of psychosocial variables on the presence, severity, and prognosis of plantar heel pain [111]. While the authors stated that there was “moderate-level” evidence associating psychosocial factors such as depression, anxiety, stress, catastrophization, and kinesiophobia to worse pain and poorer prognosis in patients with plantar heel pain, they concluded that significant heterogeneity between individual studies with regard to specific psychosocial variables and outcome measures precluded any definitive conclusions from being drawn. Further, these findings did not include interventions that specifically targeted psychological aspects and measured treatment outcomes of plantar fasciitis.

## 4. Discussion

This systematic review provides a comprehensive summary of all published systematic reviews and meta-analyses published on diverse aspects of plantar fasciitis using defined criteria. Similar reviews have been published on the orthopedic topics including anterior cruciate ligament [112], rotator cuff [113], and hip arthroscopic surgery [114]. Our review suggests that despite numerous risk factors suggested, BMI was most consistently found to increase the risk of plantar fasciitis in non-athletic population. The clinical evaluation of patient history and physical examination is appropriate for evaluating for plantar fasciitis, and among imaging studies ultrasound may be most useful in evaluating outcomes and guiding interventions that require precise localization (e.g., injections).

Regarding treatment efficacy for management of plantar fasciitis, outcomes using corticosteroid, PRP, and ESWT may have the largest number of studies and most reviews concluded longer-term outcomes favored non-corticosteroid interventions. Similar to tendinopathy, plantar fasciitis has been characterized by degeneration of collagen even though inflammation may play an important role in the early disease process [7]. However, this does not mean that inflammation and degeneration represent a continuum of disease but reflect two distinct or often coexisting processes [7,115]. Therefore, corticosteroids may offer benefits in short term particularly within 1 month following treatment. Studies also support use of ultrasound guidance for more predictable treatment response with steroid injections. However, the benefits were not durable in most studies, and clinicians should be aware of potential detrimental effects, such as plantar fascia rupture.

There is evidence to support the use of PRP compared to corticosteroid or placebo, especially at longer terms such as at 3, 6, and 12 months. One of the most recent reviews by Hurley et al. [53] who limited studies evaluating PRP found benefits in terms of VAS score reduction even at 1–1.5 months. While PRP and corticosteroid can both decrease inflammation, PRP has biological regenerative properties such as augmenting cellular migration, enhancing cellular proliferation, and promoting angiogenesis [116]. Unfortunately, cytological composition of the PRP has been poorly reported throughout the studies, and different leukocyte concentrations (leukocyte-rich or leukocyte-poor) have been used [53]. While leukocyte-poor PRP was shown more promising results in a rabbit Achilles tendinopathy model [117] and human rotator cuff tendons [118], a meta-analysis found that leukocyte-rich PRP led to a greater reduction in pain scores [119]. Due to such heterogeneity in PRP preparation methods and one RCT that showed similar outcomes between whole blood and PRP, Tseng et al. justified combining PRP and whole blood in their meta-analysis and found no significant difference between autologous blood products (PRP and whole blood) and corticosteroids [54]. Nonetheless, one NMA with limited number of studies showed that autologous blood products were better than corticosteroids in providing pain relief at 3 months, and subgroup analysis showed that when autologous blood products was limited to PRP, the treatment efficacy improved [107].

ESWT appears to provide better longer-term outcomes over corticosteroid and most interventions studied. The type (focus versus radial) and energy levels of ESWT achieved across the studies in the systematic reviews did have high heterogenicity. There was conflicting evidence regarding the optimal type and energy level of ESWT, but a recent study showed that similar functional gains were seen in between radial shockwave and radial shockwave combined with focused shockwave therapies while using a standardized physical therapy protocol [120].

Among the many studies that investigating mechanical treatments, the vast majority examined the use of orthotic insoles and taping, and limited efficacy was observed over short duration. Several studies supported the use of insoles in improving pain and function, though their overall clinical impact was questionable. There appeared to be minimal differences between different types of insoles with sham conditions providing better relief in some studies. Low dye and calcaneal taping methods appeared to be effective in alleviating pain in periods of less than one week. Stretching, manual therapy, and laser, were all generally associated with positive outcomes in terms of pain and function. There was very limited evidence to support the use of foot muscle training as well as needling therapies.

Comparisons across all treatment options is challenging due to high degree of heterogenicity and outcome measures used. However, our review did identify a number of NMAs evaluating treatment outcomes for management of plantar fasciitis. Tsikopoulos et al. compared across injection therapies and identified dehydrated amniotic membrane intervention had the highest probability of being the best injection therapy within 2 months and botox injection treatment best at 6 months [108]. Across NMAs including both injection and other interventions, ESWT was consistently found to be an effective treatment option over 1–6 months [10,107,109,110].

This review identifies limitations common to systematic reviews and meta-analyses on plantar fasciitis. While “plantar heel pain” is a generalized term encompassing a broad range of pathologies such as peripheral nerve entrapment, presence of calcaneal spur, or other potential undefined contributors, the authors of the included reviews used the term interchangeably with plantar fasciitis or did not make strict distinction between plantar fasciitis and other pathologies that may contribute to symptoms or influence outcomes. Second, many studies include co-interventions of stretching, mechanical treatments or manual therapies that make it difficult to isolate the relative influence of a given intervention on improvement in pain or function. Third, while we attempted to include and analyze all systematic reviews related to plantar fasciitis, it does not mean that we covered every aspect of plantar fasciitis. There could be original studies on other aspects of plantar fasciitis that might have not been included in a systematic review. Fourth, clinical protocols may vary in both delivery of intervention of PRP or ESWT and treatments including stretching, laser, or needling therapy. Fifth, some reviews and NMA would reach conclusions on a small number of studies. Last, while the VAS score was most commonly used for pain assessment and the AOFAS score for functional evaluation, these two measures have not been validated for the use in plantar fasciitis [49]. Furthermore, other measures such as Roles and Maudsley scale or Foot Function Index have not been consistently used across studies and interventions, which make comparison of results more challenging. These limitations should be considered when reading and implementing the results of systematic reviews in clinical decision making.

### Future Research Direction

Prevalence and incidence of plantar fasciitis have been studied and the most common identified risk factor in non-athletes is higher BMI. Risk factors for plantar fasciitis in the athletic population, however, have been poorly described. Furthermore, even though some kinetic variables such as load rates have been implicated in the development of plantar fasciitis [121], kinematic variables seemed to be more evaluated in currently available systematic reviews. Additional investigations characterizing the epidemiology and risk factors for plantar fasciitis, particularly in athlete populations and on biomechanical variables, may help establish prevention strategies. While plantar fasciitis shares many pathologic characteristics with degenerative tendon pathologies [7], the evidence of strength training or stretching in treatment of plantar fasciitis is limited. Given the possible weaker foot muscle strengths and smaller foot muscle volume in the plantar fasciitis patients [26] and demonstrated effectiveness of high-load strengthening interventions in the treatment of tendinopathies [122], similar interventions may hold promise in the management of plantar fasciitis and demonstrate the need for high level study on this topic.

Cost-effectiveness studies would also advance determining appropriate treatment paradigms and improve patient outcomes and healthcare delivery. Relative efficacy and differences in costs associated with treatments influences clinical decision making and healthcare delivery. MRI and corticosteroid injection(s) are often utilized for evaluation and treatment of plantar fasciitis despite limited evidence for role in management and outcomes. In contrast, our review identified clinical outcomes in longer-term may be improved with interventions that have more direct out of pocket costs to consumers including PRP and ESWT. A single injection of PRP may cost between $500 and $2500 [123], and ESWT is also not commonly reimbursed. Notably reviews suggested low level of evidence in outcomes for surgical intervention in treating plantar fasciitis compared to less expensive non-operative treatments. Further investigations may help clarify relative benefits of non-surgical interventions that account for individual characteristics that may influence outcomes.

Heterogeneity in intervention protocols, outcome measures, and follow-up periods were widely present across the studies. Even within the same treatment, the optimal type, dose, or volume has not yet been determined. Efforts need to be made to establish standard or most efficacious protocol in treatments such as PRP or ESWT. Furthermore, a valid and reliable measure that evaluates both pain and function specifically for plantar fasciitis could be developed to make results across studies more comparable. Lastly, most studies followed patients up to less than one year. Given the chronic and relapsing nature of plantar fasciitis, future studies should aim to follow patients longer than one year.

## 5. Conclusions

This study is a comprehensive systemic summary of meta-analyses and systemic reviews on diverse topics such as the epidemiology, diagnosis, and treatment of plantar fasciitis. While the majority of reviews had high level of heterogeneity and included a small number of studies pertaining to plantar fasciitis, there was some consensus on certain topics, such as BMI as a risk factor for plantar fasciitis and ESWT and PRP both appearing to be safe and effective in longer-term outcomes. Evidence on topics such as the epidemiology, exercise therapy, or cost-effectiveness of treatment options for plantar fasciitis are limited compared to other topics and may warrant future research.

## Data Availability

Data Sharing not applicable.

## Supplementary Materials

The following are available online at https://www.mdpi.com/article/10.3390/life11121287/s1, Figure S1: Search Strategy.

## Appendix A

**Table A1:** Methodological quality evaluation of 96 systematic reviews included in this study.

Author/Year	Item 1	Item 2	Item 3	Item 4	Item 5	Item 6	Item 7	Item 8	Item 9	Item 10	Item 11	Item 12	Item 13	Item 14	Item 15	Item 16	Overall Assessment
Agyekum 2015	Yes	No	No	No	No	No	No	No	No	No	N/A	N/A	No	No	N/A	No	Critically low
Al-Abbad 2020	Yes	Yes	No	Partial Yes	Yes	Yes	No	Partial Yes	Yes	No	Yes	No	Yes	Yes	Yes	Yes	Low
Al-Boloushi 2019	Yes	Yes	No	Partial Yes	Yes	Yes	No	Partial Yes	Yes	No	N/A	N/A	No	Yes	N/A	Yes	Critically low
Alkhatib 2020	Yes	No	No	Partial Yes	Yes	Yes	No	Partial Yes	Yes	No	Yes	No	No	Yes	Yes	Yes	Critically low
Andia 2015	Yes	No	Yes	No	No	No	No	Partial Yes	No	No	N/A	N/A	No	Yes	N/A	Yes	Critically low
Aqil 2013	Yes	No	No	Partial Yes	Yes	Yes	No	Partial Yes	Partial Yes	No	Yes	No	No	Yes	No	Yes	Critically low
Assad 2016	Yes	No	No	Partial Yes	No	No	No	Partial Yes	No	No	N/A	N/A	No	No	N/A	Yes	Critically low
Atkins 1999	Yes	No	Yes	Partial Yes	No	Yes	No	Partial Yes	Partial Yes	No	N/A	N/A	Yes	Yes	N/A	Yes	Critically low
Babatunde 2019	Yes	Yes	No	Partial Yes	Yes	Yes	Yes	Yes	Yes	No	Yes	Yes	Yes	Yes	Yes	Yes	Moderate
Brantingham 2012	Yes	No	Yes	Partial Yes	Yes	Yes	No	Partial Yes	Yes	No	N/A	N/A	Yes	No	N/A	Yes	Critically low
Butterworth 2012	Yes	No	Yes	Partial Yes	Yes	Yes	Yes	Partial yes	Partial Yes	No	N/A	N/A	Yes	Yes	N/A	Yes	Low
Chang 2012	Yes	No	Yes	Partial Yes	Yes	Yes	No	Parial Yes	Partial Yes	No	Yes	Yes	Yes	Yes	No	Yes	Critically low
Chen 2018	Yes	No	No	Partial Yes	Yes	Yes	No	Partial Yes	Partial Yes	No	Yes	No	No	No	No	Yes	Critically low
Chen 2019	Yes	No	Yes	Partial Yes	Yes	No	No	Partial Yes	Partial Yes	No	Yes	Yes	Yes	Yes	Yes	Yes	Critically low
Chiew 2016	Yes	No	No	Partial Yes	Yes	Yes	No	Partial Yes	Partial Yes	No	N/A	N/A	Yes	Yes	N/A	Yes	Critically low
Clar 2014	Yes	No	No	Partial Yes	Yes	Yes	No	Partial Yes	Yes	Yes	N/A	N/A	Yes	Yes	N/A	Yes	Critically low
Clark 2012	Yes	Yes	No	Partial Yes	No	No	No	Partial Yes	Partial Yes	Yes	N/A	N/A	Yes	Yes	N/A	Yes	Low
Clijsen 2012	Yes	No	No	Partial Yes	Yes	Yes	No	Partial Yes	Yes	No	Yes	No	Yes	Yes	N/A	Yes	Critically low
Cotchett 2010	Yes	No	Yes	Partial Yes	Yes	Yes	Yes	Partial Yes	Yes	No	N/A	N/A	Yes	Yes	N/A	Yes	Low
David 2017	Yes	Yes	Yes	Yes	Yes	Yes	Yes	Yes	Yes	Yes	Yes	Yes	Yes	Yes	Yes	Yes	High
Dizon 2013	Yes	No	Yes	Partial Yes	No	No	No	Partial Yes	Yes	No	Yes	Yes	Yes	No	No	Yes	Critically low
Dos Santos 2019	Yes	Yes	No	Partial Yes	Yes	Yes	No	Partial Yes	Yes	No	Yes	Yes	Yes	Yes	No	Yes	Critically low
Drake 2018	Yes	Yes	No	Partial Yes	Yes	Yes	No	Partial Yes	Yes	No	N/A	N/A	Yes	Yes	N/A	Yes	Low
Franceschi 2014 (PRP)	Yes	No	No	Partial Yes	Yes	Yes	No	Partial Yes	No	No	N/A	N/A	No	Yes	N/A	Yes	Critically low
Franceschi 2015	Yes	No	Yes	Partial Yes	Yes	Yes	No	Partial Yes	No	No	N/A	N/A	No	Yes	N/A	Yes	Critically low
Franchini 2018	Yes	No	No	Partial Yes	Yes	Yes	No	Partial Yes	Yes	No	Yes	No	Yes	Yes	No	Yes	Critically low
Fraser 2018	Yes	Yes	No	Partial Yes	No	No	No	Partial Yes	Yes	No	N/A	N/A	Yes	Yes	N/A	Yes	Critically low
Fusini 2017	Yes	No	No	Partial Yes	Yes	No	No	Partial Yes	No	No	N/A	N/A	No	No	N/A	No	Critically low
Hamstra-Wright 2021	Yes	No	No	Partial Yes	Yes	Yes	No	Partial Yes	No	Yes	Yes	No	No	Yes	No	Yes	Critically low
Hawke 2008	Yes	No	Yes	Yes	Yes	Yes	Yes	Yes	Yes	No	Yes	Yes	Yes	Yes	No	Yes	Critically low
He 2017	Yes	No	No	Partial Yes	Yes	No	Yes	Partial Yes	Yes	No	Yes	Yes	Yes	Yes	Yes	Yes	Low
Healy 2018	Yes	Yes	Yes	Partial Yes	Yes	Yes	Yes	Partial Yes	Yes	No	N/A	N/A	Yes	Yes	N/A	Yes	High
Hohmann 2020	Yes	No	No	Partial Yes	Yes	No	No	Partial Yes	Yes	No	Yes	No	Yes	Yes	Yes	Yes	Critically low
Hsiao 2015	Yes	No	No	Partial Yes	Yes	Yes	No	Partial Yes	Partial Yes	No	Yes	No	No	Yes	Yes	Yes	Critically low
Huang 2020	Yes	Yes	No	Partial Yes	Yes	No	No	Partial Yes	Yes	Yes	Yes	Yes	Yes	Yes	No	Yes	Critically low
Huffer 2017	Yes	Yes	No	Partial Yes	No	No	No	Partial Yes	Partial Yes	No	N/A	N/A	No	Yes	N/A	Yes	Critically low
Hurley 2020	Yes	No	No	Partial Yes	Yes	Yes	No	Patial Yes	Yes	No	Yes	No	Yes	Yes	No	Yes	Critically low
Irving 2006	Yes	No	Yes	Partial Yes	Yes	No	No	Partial Yes	Partial Yes	No	N/A	N/A	No	Yes	N/A	No	Critically low
Lee 2009	Yes	No	No	Partial Yes	Yes	No	No	Partial Yes	Yes	No	No	No	Yes	Yes	No	Yes	Critically low
Li 2013	Yes	No	No	Partial Yes	Yes	Yes	No	Partial Yes	Partial Yes	No	Yes	Yes	Yes	Yes	No	Yes	Critically low
Li 2014	Yes	No	No	Partial Yes	Yes	Yes	No	Partial Yes	Yes	No	Yes	No	Yes	Yes	Yes	Yes	Critically low
Li 2015	Yes	No	No	Partial Yes	No	Yes	No	Partial Yes	Yes	No	Yes	Yes	Yes	Yes	Yes	No	Critically low
S Li 2018	Yes	No	No	Partial Yes	Yes	Yes	No	Partial Yes	Yes	No	Yes	No	No	Yes	Yes	Yes	Critically low
X Li 2018	Yes	Yes	No	Partial Yes	Yes	Yes	No	Partial Yes	Yes	Yes	Yes	Yes	Yes	Yes	No	Yes	Critically low
H Li 2018	Yes	No	No	Partial Yes	Yes	No	No	Yes	No	No	Yes	No	No	Yes	Yes	Yes	Critically low
Li 2019	Yes	No	No	Partial Yes	Yes	Yes	No	Partial Yes	Yes	No	Yes	Yes	Yes	Yes	No	Yes	Critically low
Ling 2018	Yes	No	No	Partial Yes	No	Yes	No	Partial Yes	Yes	No	Yes	Yes	Yes	Yes	Yes	Yes	Critically low
Lou 2017	Yes	No	No	Partial Yes	Yes	Yes	No	Partial Yes	Yes	No	Yes	Yes	Yes	Yes	No	Yes	Critically low
Malahias 2020	Yes	No	No	Partial Yes	Yes	Yes	No	Partial Yes	No	No	N/A	N/A	No	Yes	N/A	Yes	Critically low
Mao 2019	Yes	No	No	Partial Yes	Yes	No	No	Partial Yes	No	No	No	No	No	Yes	No	Yes	Critically low
McMillan 2009	Yes	Yes	No	Partial Yes	No	Yes	Yes	Partial Yes	Yes	No	Yes	No	Yes	Yes	Yes	Yes	Moderate
Mendes 2020	Yes	Yes	No	Partial Yes	Yes	No	No	Partial Yes	Yes	No	N/A	N/A	No	No	N/A	Yes	Critically low
Mischke 2017	Yes	No	No	Partial Yes	Yes	No	No	Partial Yes	Yes	No	N/A	N/A	Yes	Yes	N/A	Yes	Critically low
Mohammed 2020	Yes	Yes	No	Partial Yes	No	Yes	No	Partial Yes	Yes	No	Yes	No	Yes	Yes	Yes	Yes	Low
Mohseni-Bandpei 2014	Yes	No	No	Partial Yes	Yes	No	No	Partial Yes	Partial Yes	No	N/A	N/A	No	No	N/A	No	Critically low
Mousavi 2019	Yes	No	No	Partial Yes	Yes	No	No	Partial Yes	Yes	No	Yes	No	No	No	No	Yes	Critically low
Ogden 2002	Yes	No	No	No	No	No	No	No	No	No	No	No	No	No	No	No	Critically low
Osborne 2019	Yes	No	No	Partial Yes	Yes	Yes	No	Partial Yes	Yes	No	Yes	No	Yes	Yes	No	Yes	Critically low
Petragila 2017	Yes	No	No	Partial Yes	No	Yes	No	Partial Yes	No	No	N/A	N/A	No	No	N/A	No	Critically low
Philips 2017	Yes	No	No	Partial Yes	Yes	Yes	No	Partial Yes	Yes	No	N/A	N/A	Yes	Yes	N/A	Yes	Critically low
Piper 2016	Yes	Yes	No	Partial Yes	Yes	Yes	No	Partial Yes	Yes	No	N/A	N/A	No	No	N/A	Yes	Critically low
Podolsky 2015	Yes	No	No	Partial Yes	No	No	No	Partial Yes	Yes	No	N/A	N/A	No	Yes	N/A	Yes	Critically low
Radwan 2016	Yes	No	No	Partial Yes	Yes	No	No	Partial Yes	Yes	Yes	N/A	N/A	No	Yes	N/A	No	Critically low
Rasenberg 2018	Yes	Yes	No	Partial Yes	Yes	Yes	No	Yes	Yes	No	Yes	Yes	Yes	Yes	No	Yes	Critically low
Roerdink 2017	Yes	No	No	Partial Yes	Yes	Yes	No	Yes	No	No	N/A	N/A	Yes	Yes	N/A	Yes	Critically low
Salvioli 2017	Yes	Yes	No	Partial Yes	Yes	Yes	No	Partial Yes	Yes	No	Yes	No	Yes	Yes	No	Yes	Critically low
Sanderson 2015	Yes	No	No	Partial Yes	No	No	Yes	Partial Yes	Partial Yes	No	N/A	N/A	Yes	Yes	N/A	Yes	Low
Schuitema 2020	Yes	No	No	Partial Yes	Yes	No	No	Partial Yes	Yes	Yes	N/A	N/A	Yes	Yes	N/A	Yes	Critically low
Sheth 2012	Yes	No	No	Partial Yes	Yes	Yes	No	Partial Yes	Partial Yes	Yes	Yes	No	Yes	Yes	Yes	Yes	Critically low
Singh 2017	Yes	Yes	No	Partial Yes	No	Yes	No	Partial Yes	Partial Yes	No	Yes	Yes	Yes	Yes	No	Yes	Critically low
Siriphorn 2020	Yes	Yes	No	Partial Yes	No	No	No	Partial Yes	Yes	No	Yes	No	Yes	Yes	Yes	Yes	Low
Speed 2013	Yes	No	Yes	Partial Yes	No	No	No	Partial Yes	No	No	N/A	N/A	No	Yes	N/A	Yes	Critically low
Sun 2017	Yes	No	No	Partial Yes	No	Yes	Yes	Partial Yes	Yes	No	Yes	Yes	Yes	Yes	No	Yes	Critically low
Sun 2020	Yes	No	Yes	Partial Yes	Yes	Yes	No	No	Yes	No	Yes	No	Yes	No	No	Yes	Critically low
Sutton 2016	Yes	Yes	No	Partial Yes	Yes	Yes	No	Partial Yes	Yes	No	N/A	N/A	Yes	Yes	N/A	Yes	Low
Sweeting 2011	Yes	No	No	Partial Yes	Yes	Yes	No	Partial Yes	Yes	No	N/A	N/A	Yes	Yes	N/A	Yes	Critically low
Thiagarajah 2017	Yes	No	No	Partial Yes	No	No	No	Partial Yes	Yes	No	N/A	N/A	No	Yes	N/A	No	Critically low
Thomson 2005	Yes	No	No	Partial Yes	Yes	Yes	Yes	Partial Yes	Yes	Yes	Yes	Yes	Yes	Yes	Yes	Yes	Low
Tseng 2020	Yes	Yes	Yes	Partial Yes	Yes	No	No	Partial Yes	Yes	No	Yes	Yes	Yes	Yes	No	Yes	Critically low
Tsikopoulos 2016 (AWB)	Yes	Yes	No	Partial Yes	Yes	Yes	Yes	Partial Yes	Yes	Yes	Yes	Yes	Yes	Yes	No	Yes	Low
Tsikopoulos 2016 (NMA))	Yes	Yes	No	Partial Yes	Yes	Yes	Yes	Partial Yes	Yes	Yes	Yes	Yes	Yes	Yes	Yes	Yes	High
Uden 2011	Yes	No	Yes	Partial Yes	Yes	Yes	Yes	Partial Yes	Yes	No	N/A	N/A	Yes	No	N/A	Yes	Low
Van de Water 2010	Yes	No	No	Partial Yes	Yes	Yes	Yes	Partial Yes	Yes	No	N/A	N/A	Yes	Yes	N/A	Yes	Low
Van Leeuwen 2016	Yes	Partial Yes	Yes	Partial Yes	Yes	Yes	No	Yes	Yes	No	Yes	No	Yes	Yes	Yes	Yes	Low
Vannini 2014	Yes	No	No	No	No	No	No	Partial Yes	No	No	N/A	N/A	No	Yes	N/A	Yes	Critically low
Waclawski 2015	Yes	Partial Yes	No	Partial Yes	Yes	No	Partial Yes	Partial Yes	Yes	No	N/A	N/A	Yes	Yes	N/A	Yes	Moderate
Wang 2019	Yes	No	No	Partial Yes	Yes	Yes	No	Partial Yes	Yes	No	Yes	No	Yes	Yes	No	Yes	Critically low
Wang 2019 (Laser)	Yes	No	No	Partial Yes	No	Yes	No	Partial Yes	Yes	No	Yes	No	No	No	Yes	Yes	Critically low
Whittaker 2017	Yes	No	No	Partial Yes	Yes	Yes	No	Partial Yes	Yes	No	Yes	Yes	Yes	Yes	Yes	Yes	Critically low
Whittaker 2019	Yes	Yes	No	Partial Yes	Yes	Yes	No	Partial Yes	Yes	No	Yes	Yes	Yes	Yes	Yes	Yes	Low
Woitzik 2015	Yes	Yes	No	Partial Yes	Yes	Yes	No	Yes	Yes	No	N/A	N/A	Yes	Yes	N/A	Yes	Low
Xiong 2019	Yes	No	No	Partial Yes	Yes	Yes	No	Partial Yes	Yes	No	Yes	No	No	Yes	No	Yes	Critically low
Yang 2017	Yes	No	No	Partial Yes	No	Yes	No	Partial Yes	Yes	No	Yes	No	No	Yes	No	Yes	Critically low
Yin 2014	Yes	No	No	Partial Yes	No	Yes	No	Partial Yes	Yes	No	Yes	No	Yes	Yes	Yes	Yes	Critically low
Yu 2016	Yes	Yes	Yes	Partial Yes	Yes	Yes	Yes	Yes	Yes	No	N/A	N/A	Yes	Yes	N/A	Yes	High
Yu 2020 (PRP)	Yes	No	No	Partial Yes	No	No	No	Partial Yes	Yes	No	Yes	Yes	Yes	Yes	No	Yes	Critically low
