# Supplementary material for: A Systematic Review of Systematic Reviews on the Epidemiology, Evaluation, and Treatment of Plantar Fasciitis
Source: Life (Basel). 2021 Nov 24;11(12):1287. doi: 10.3390/life11121287 (PMC8705263; doi:10.3390/life11121287)
Supplement: Supplementary file 1 [file life-11-01287-s001.zip › life-1450837-supplementary.pdf]

Search Strategy

PUBMED
Search terms: "Fasciitis, Plantar"[Mesh] OR plantar heel pain[tw] OR plantar fasci*[tw]
AND
“systematic review”[pt] OR systematic[ti] OR “meta-analysis”[pt] OR meta*[tw]

Total Results: 390

EMBASE
('plantar fasciitis'/exp OR 'plantar fasciopathy'/exp OR 'plantar heel pain'/exp OR ‘plantar fasci*’:ab,ti,kw)
AND
'systematic review'/exp OR 'meta analysis'/exp OR systematic: ab,ti,kw OR meta*:ab,ti,kw
AND
[embase]/lim OR [embase classic]/lim

Total Results: 395

Web of Science
“plantar fasciitis” OR “plantar fasciopathy” OR “plantar heel pain” OR “plantar fasci*”
AND
“Systematic review” OR “meta-analysis” OR systematic OR meta*

Total Results: 246

Cochrane Library
1. MeSH Fasciitis, Plantar
2. ‘plantar fasci*’ OR ‘plantar heel pain’ or ‘heel spur’
3. ‘systematic review’ OR ‘meta-analysis’
4. #1 OR #2
5. #4 AND #3

Total Results: 43
